# Genome sequence of the plant growth promoting endophytic yeast *Rhodotorula graminis* WP1

**DOI:** 10.3389/fmicb.2015.00978

**Published:** 2015-09-17

**Authors:** Andrea Firrincieli, Robert Otillar, Asaf Salamov, Jeremy Schmutz, Zareen Khan, Regina S. Redman, Neil D. Fleck, Erika Lindquist, Igor V. Grigoriev, Sharon L. Doty

**Affiliations:** ^1^Department for Innovation Biological, Agro-Food and Forest System, University of TusciaTuscia, Italy; ^2^U.S. Department of Energy Joint Genome InstituteWalnut Creek, CA, USA; ^3^HudsonAlpha Institute for BiotechnologyHuntsville, AL, USA; ^4^School of Environmental and Forest Sciences, University of WashingtonSeattle, WA, USA; ^5^Adaptive Symbiotic TechnologiesSeattle, WA, USA

**Keywords:** symbiosis, endophytes, populus, plant-microbe interactions, endophytic yeast, endophyte genomics, microbiome, phytobiome

## Background

Here we present the genome sequence of *Rhodotorula graminis* WP1, a pink-pigmented, encapsulated yeast strain belonging to the Basidiomycota phylum that was isolated from within stems of *Populus trichocarpa* growing in its native riparian environment alongside the Snoqualmie River in Western Washington state(Xin et al., 2009). Although numerous bacterial endophytes, the microorganisms living fully within plants, have been characterized, relatively few endophytic yeast strains have been studied (Doty, 2011). The genome of strain WP1 was the first endophytic yeast to be sequenced, and has been included in several genomic analyses (Spatafora et al., 2013; Nagy et al., 2014; Pendleton et al., 2014). Although originally isolated from poplar, WP1 has a broad host range, providing growth benefits not only to poplar (Knoth et al., 2014; Khan et al. in review) but also to grasses and agricultural crop species (Khan et al., 2012; Knoth et al., 2013). Strain WP1 improves plant vigor and has useful biochemical properties (Doty, 2014) including the ability to ferment both pentoses and hexoses and to degrade fermentation inhibitors (Xu et al., 2011). Genomic analysis of bacterial endophytes has revealed certain features in common including genes for phytohormone synthesis, adhesion, colonization, stress reduction, and iron and phosphate acquisition (Fouts et al., 2008; Taghavi et al., 2010; Sessitsch et al., 2012; Witzel et al., 2012). We analyzed the genome of WP1 with a focus on genes potentially involved in plant-microbe interactions.

## Methods

### DNA purification

*Rhodotorula graminis* strain WP1 was isolated from surface-sterilized shoot cuttings of poplar (*P. trichocarpa*) collected at the Three Forks Natural Area in King County, WA in the riparian zone of the Snoqualmie River (+47° 31′ 14.30″, −121° 46′ 28.32″) in August 2002, and glycerol stocks were frozen at −70°C. All subsequent studies were done using samples from these cryo-stocks. For DNA extraction, WP1 was grown in 300 ml YPD (yeast extract, peptone, dextrose) broth, pelleted, washed twice in sterile water, frozen, and freeze-dried. Cells were lysed with glass beads and the genomic DNA purified as previously described (Pitkin et al., 1996).

### RNA extraction

A single WP1 colony from solid NFMS [Nitrogen-Free Murashige and Skoog medium (Murashige and Skoog, 1962); Caisson] was used to inoculate 100 ml of liquid NFMS, and grown with agitation (200–250 rpm) for 24 h at RT. Cells were harvested and resuspended in 100 ml YPD broth and grown with agitation at RT. After 2 days, cells were centrifuged at 1300 × g and washed twice with NFMS medium. Cell density was adjusted to an OD_600_ 0.4 in fresh NFMS and grown with agitation (200–250 rpm) at RT until the cell density reached an OD_600_ of 1.2. Cells were centrifuged at 1300 × g, washed twice in NFMS media, frozen at −80°C overnight, and lyophilized. Cells were ground in liquid nitrogen until a fine powder was achieved, and RNA extracted using standard protocols as suggested by the manufacturers of the reagent TRlzol (Life Technologies).

### Genome sequencing

The *Rhodotorula graminis* strain WP1 genome was sequenced by the Joint Genome Institute (JGI) using the Sanger whole genome shotgun approach. Three (3, 6, and 33.8 kb insert size) libraries were sequenced. Sequenced reads were QC filtered for vector sequence, mitochondria, unanchored rDNA, and assembled with the Arachne assembler (Jaffe et al., 2003). The 21,013,998 bp assembly resulted in 26 scaffolds comprising 323 contigs with average read depth coverage of 8.55x (Table 1).

**Table 1:** **Genome assembly statistics for *R. graminis* strain WP1**.

Assembly length, bp	21,031,998
Number of scaffolds	26
Scaffold N50	7
Scaffold L50, bp	1,420,730
Number of contigs	323
Contig N50	38
Contig L50, bp	167,432
%GC	67.76

### Transcriptome sequencing

For analysis of the *R. graminis* transcriptome, polyA mRNA was used to construct cDNA libraries and these were sequenced using the Roche-454 GS-FLX platform. The 1.9 million reads were filtered and screened for quality and contamination and were assembled into contigs using Newbler (v2.3-PreRelease-6/30/2009) with default parameters.

### Genome annotation

The *R. graminis* genome assembly was annotated using the JGI Annotation Pipeline (Grigoriev et al., 2006), which combines several gene prediction and functional annotation methods, and integrates the annotated genome into JGI web-based resource for fungal comparative genomics, MycoCosm (http://jgi.doe.gov/fungi) (Grigoriev et al., 2014). Before gene prediction, assembly scaffolds were masked using RepeatMasker (Smit et al., 2010), RepBase library (Jurka et al., 2005), and the most frequent (>150 times) repeats recognized by RepeatScout (Price et al., 2005). The following combination of gene predictors was run on the masked assembly: ab initio including Fgenesh (Salamov and Solovyev, 2000) and GeneMark (Ter-Hovhannisyan et al., 2008); homology-based including Fgenesh+ (Salamov and Solovyev, 2000) and Genewise (Birney and Durbin, 2000) seeded by BLASTx alignments against the NCBI NR database; and transcriptome-based using Fgenesh package. In addition to protein-coding genes, tRNAs were predicted using tRNAscan-SE (Lowe and Eddy, 1997). For each genomic locus, the best representative gene model was selected based on a combination of protein homology and transcriptome support. All predicted proteins were functionally annotated using SignalP (Nielsen et al., 1997) for signal sequences, TMHMM (Melén et al., 2003) for transmembrane domains, interProScan (Quevillon et al., 2005) for integrated collection of functional and structural protein domains, and protein alignments to NCBI nr, SwissProt (Boeckmann et al., 2003), KEGG (Kanehisa et al., 2004) for metabolic pathways, and KOG (Koonin et al., 2004) for eukaryotic clusters of orthologs. InterPro and SwissProt hits were used to map Gene Ontology terms (Ashburner et al., 2000).

## Results

### Genome characteristics

The 21.01 Mbp genome of *Rhodotorula graminis* strain WP1 was assembled in 28 scaffolds and 323 contigs with 1.13% in scaffold gaps (Table 1). The genome contains 3.63% repetitive DNA and 7283 predicted genes, supported by transcriptomics and homology to proteins from other fungi (Table 2). Three thousand five hundred and fifty-two predicted proteins form 929 multigene families based on MCL clustering, the largest of which include protein kinases, transporters, and transcription factors (http://genome.jgi.doe.gov/Rhoba1_1).

**Table 2:** **Gene content of *R. graminis* strain WP1**.

	**Annotation results**	
Number of genes	7283	100%
Avg. gene length, bp	2126	
Avg. protein length, aa	501	
Avg. exon length, bp	254	
Avg. intron length, bp	105	
Avg. exons per gene	6.2	
**PROTEINS WITH:**		
Similarity to KEGG	6384	88%
Similarity to KOG	5910	81%
Similarity to Swissprot	5989	82%
Similarity to NCBI NR	5724	79%
Pfam domain	4545	62%
Complete CDS	6415	88%
Transmembrane helices	1283	18%
Signal peptide	1626	22%

*R. graminis* has one of the highest GC-rich genomes (67%) among the all publicly available fungal genomes and while expectedly its intron content is also GC-rich, a significant imbalance in the C (42.7%) and G (23%) content of introns is surprising. This 19.7% deviation is the largest asymmetry in C/G content observed among the introns of all fungal genomes in MycoCosm (Grigoriev et al., 2014), where the average asymmetry is 1.8 ± 3.1%, and the second highest asymmetry of 7.3% (29.5% C and 22.2% G) was identified in the related Pucciniomycotina yeast *Sporobolomyces roseus.* Additionally, an extended donor splice consensus observed in introns of *R. graminis* is likely due to the high C content of introns. Most eukaryotes have a conserved consensus at positions +1 to +6 of introns (Rogozin and Milanesi, 1997; Bhasi et al., 2007). In *R. graminis*, positions +7 to +10 have a predominantly C nucleotide, with positions +3 and +4 also having stronger consensus relative to what is observed in other basidiomycetes.

### Phytohormones, volatile organic compounds (VOCs), capsule production and small secreted proteins (SSPs)

A common feature of endophytic bacteria and fungi is the production of phytohormones (Hardoim et al., 2008; Bulgarelli et al., 2013; Sukumar et al., 2013; Duca et al., 2014). Many of the beneficial plant-associated microorganisms that produce auxin rely on plant-exuded tryptophan as the precursor for biosynthesis of the auxin, indole-3-acetic acid (IAA) (Hardoim et al., 2008). Since auxins stimulate plant growth and there is a higher prevalence of auxin-producing microorganisms within plants than in the rhizosphere, it has been proposed that the plant host environment selects for endophytes with this trait (Patten and Glick, 2002). Genomic analysis of several bacterial endophytes, including *Enterobacter* from poplar stems (Taghavi et al., 2010), *Gluconacetobacter diazotrophicus* from sugarcane (Bertalan et al., 2009), and the endophyte community of rice (Sessitsch et al., 2012) provided evidence of multiple microbial pathways for auxin biosynthesis. Although less is known about endophytic yeast than endophytic bacteria, there is evidence of plant growth promotion by auxin-producing yeasts (Doty, 2011). Eight of the *Williopsis saturnus* endophytic yeast strains of maize roots produced the auxins IAA and IPYA (Nassar et al., 2005). Since WP1 has strong root-promoting activity on recalcitrant poplar clones (Doty, unpublished) and overall plant growth-promoting activity (Khan et al., 2012; Knoth et al., 2014), we analyzed the genome for evidence of auxin and other phytohormone biosynthesis capabilities, putative effectors involved in plant-microbe interaction, and antitoxin systems.

The WP1 genome lacks the standard genes encoding for proteins involved in the biosynthesis of IAA via indole-3-pyruvate (KEGG Entry: R00677; R00684) and indole-3-acetamide (KEGG Entry: R00679). However, three putative proteins, an aromatic-L-amino-acid decarboxylase (Protein ID: 35429), a monoamine oxidase (Protein ID: 54216) and an indol-3-acetaldehyde dehydrogenase (Protein ID: 14581) can be involved in the conversion of L-tryptophan to IAA via tryptamine. In this pathway, trp is first decarboxylated to tryptamine (KEGG Entry: R00685) which is subsequently oxidized into indole-3-acetaldehyde and then to IAA through two consecutive oxidation steps carried out by an amine oxidase (KEGG Entry: R02173) and an aldehyde dehydrogenase, respectively (KEGG Entry: R02678). Furthermore, two putative hydrolases (Protein ID: 34153; 66162) belonging to the nitrilase superfamily, which are important for the microbial colonization of plants due to their role in nitriles detoxification and utilization of plant nitriles as carbon and nitrogen source (Howden and Preston, 2009; Howden et al., 2009), can also convert the indole-3-acetonitrile (IAN) into IAA (KEGG Entry: R03093). However, IAN synthesis occurring in microbes is still unclear (Fu and Wang, 2011).

From research for functional domains, 15 proteins in WP1 were annotated as containing a 2OG-Fe(II) oxygenase domain (IPR005123) which are primarily involved in the biosynthesis of gibberellins and other plant hormones (Prescott and Lloyd, 2000; Zhao et al., 2013; Farrow and Facchini, 2014). WP1 was shown to produce the phytohormones GA3, IAA, JA, ABA, and Br *in vitro* (Khan et al. in review).

*R. graminis* WP1 has a set of genes involved in the synthesis of (R)-acetoin and (R,R)-2,3-butandiol, two well-known VOCs that increase resistance to plant pathogens and also act as growth promoting factors (Johnston-Monje and Raizada, 2011; D'Alessandro et al., 2014; Taghavi et al., 2015). In WP1, a putative metabolic pathway which leads to the synthesis of (R)-acetoin and (R,R)-2,3-butandiol starts with the decarboxylation of pyruvate into 2-acetolactate (KEGG Entry: R00006) by a putative acetolactate synthase (Protein ID: 32290; 35922). Under aerobic conditions, the synthesis of (R,R)-2,3-butandiol (KEGG Entry: R02946) from (R)-acetoin by two putative NADH-dependent dehydrogenase (Protein ID: 39181; 46342) occurs through the spontaneous decarboxylation of 2-acetolactate into (R)-acetoin (Atsumi et al., 2009).

In order to offer a more in depth overview about the presence of putative SSPs (aminoacidic seq. < 300) encoding genes, as supposed effectors involved in plant-microbe interactions (Rafiqi et al., 2013), we data mined the annotation file “SignalP” as described in Pendleton et al. (2014) but with slight modification. The following file, accessible from the download section through the sub-directory path: Files > Annotation > Filtered Models > Functional Annotation > Signalp, were analyzed through the TargetP 1.1 Server (Emanuelsson et al., 2000). Among 208 putative SSPs, two coding sequences (Protein ID: 43059 and 46692) for extracellular membrane proteins with a cysteine-rich domain (CFEM domain: IPR008427) were detected. Moreover, through the Search tool, three secreted proteins (Protein ID: 55573, 55528, and 50767) with a Carbohydrate-binding WSC domain (IPR013994) were also detected.

Inoculation of roots with WP1 results in colonization of the plant, including the shoots, possibly through the formation of a yeast-form biofilm (unpublished data). Unlike bacteria that could colonize plants using flagella, most yeasts, just like filamentous fungi, may colonize, and interact with the plant through a filamentous form. The passage from a yeast-form to a filamentous stage can be triggered by a wide range of environmental stimuli or as a result of the interactions with other microorganisms within multispecies biofilms (Lengeler et al., 2000). In a study on the sexuality and life cycle in *Rhodotorula glutinis* strains, the capability to form filamentous mycelia was reported (Banno, 1967). Although we have not seen a filamentous form of WP1 under culture conditions, we cannot exclude the possibility that WP1 is able to form mycelia under specific environmental conditions.

**Figure 1:**
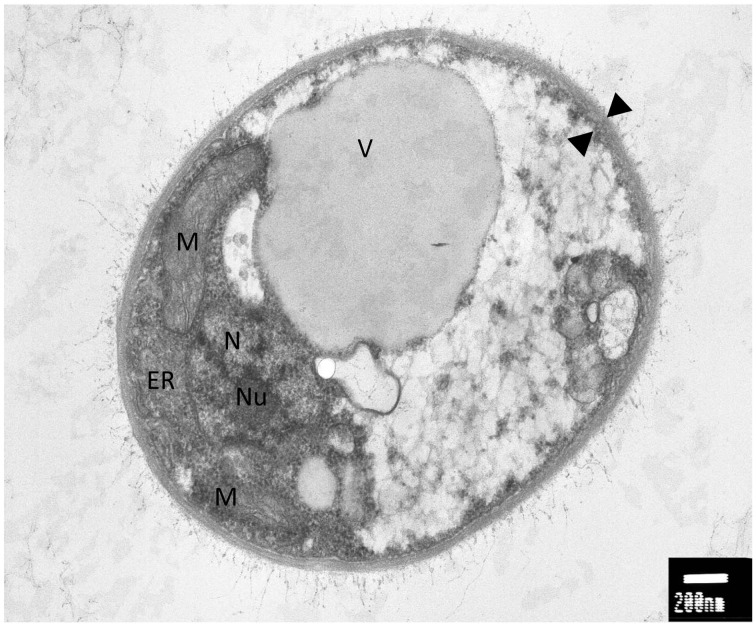
**Electron microscopy analysis of *Rhodotorula graminis* strain WP1; M, mitochondria; ER, endoplasmic reticulum; N, nucleus; Nu, nucleulus; V, vacuole; black triangles, capsule**. Photo credit: Prof. Jimmie Lara, Department of Microbiology, University of Washington.

An interesting feature of WP1 is the presence of a polysaccharidic capsule that surrounds the cellular body (Figure 1). A well-studied encapsulated yeast is represented by *Cryptococcus neoformans*, an opportunistic pathogen that causes meningoencephalitis in immunocompromised patients (Mitchell and Perfect, 1995). *C. neoformans* is ubiquitous in nature and the survival under different environmental conditions can be due to his biofilmogenic property. Within biofilms, microbial cells not only have an increased resistance and tolerance against a wide range of biotic and abiotic stress but also, under specific physiological and environmental conditions, can disperse and colonize new ecological niches (Ramage et al., 2009). The capability of *C. neoformans* to form a biofilm is dependent on the presence of the capsule. Deletion mutants in cap59 and cap10, two genes involved in the capsule synthesis and virulence, are unable to form biofilms, implicating that this structure exerts an important role in the adhesion and subsequent formation of cells aggregates (Garcia-Rivera et al., 2004; Martinez and Casadevall, 2005). Since a putative CAP59 (Protein ID: 4796) and CAP10 (Protein ID: 7100) were detected in WP1, the genome sequence of non-pathogenic encapsulated yeast would be interesting for a comparative analysis between the capsule synthesis in WP1 and *C. neoformans*.

Finally, the WP1 genome was data-mined for functional domains related to notable antitoxin systems. A set of antitoxin systems including multi antimicrobial extrusion protein (MATE), multidrug resistance efflux transporters (EmrE) and putative proteins with a multidrug resistance protein MdtG (IPR001958) domain was observed in the WP1 genome. The WP1 capsule in conjunction with these antitoxin systems represent important features that may be important for competition against other endophytes, plant colonization, and survival under different biotic and abiotic stresses.

### Other symbiotic traits

Endophytes often share in common sets of genes that are thought to confer symbiotic abilities (Sessitsch et al., 2012; Bulgarelli et al., 2013). These beneficial genes could be acquired by horizontal gene transfer (HGT) (Taghavi et al., 2005; Aminov, 2011). There has been some indication of HGT between bacteria and fungi (Marcet-Houben and Gabaldon, 2010). However, when compared via BLAST against other databases of endophyte-associate genes (Sessitsch et al., 2012), WP1 shares only minimal homology with a few highly conserved bacterial endophyte genes such as catalase (Protein ID: 47984), as well as a gene involved in cofactor-A transport (Protein ID: 66591), and a glutathione S-transferase family protein (Protein ID: 54692). All of these matches, however, are all core metabolism/housekeeping proteins, and had less than 50% identity to the bacterial query proteins. Additionally, the WP1 genome was run through an in-house pipeline designed to identify genes acquired via transdomain HGT (Thomas, unpublished), but the results were negative. This lack of significant evidence of HGT could suggest that this endophytic yeast evolved most of its plant interaction and symbiosis genes independently from its bacterial counterparts. The well-studied symbiotic Basidiomycota, *Laccaria bicolor*, begins the colonization of poplar roots using an array of effector proteins known as mycorrhiza-induced-cysteine-rich SSPs (MiSSPs) (Martin et al., 2008). A domain of the WP1 mRNA splicing factor Prp8 (ProteinID 49541) is partially homologous to the *L. bicolor* LbMiSSP17 effector protein (Protein ID: 332226). Otherwise, no homology with the other 22 *L. bicolor* MiSSPs exists. Despite the fact that both WP1 and *L. bicolor* are both Basidiomycetes which symbiotically colonize poplar, this lack of homology could suggest WP1 uses an entirely different signaling pathway to communicate with the host.

## Conclusions

There is a growing interest in the plant microbiome and its impacts on plant health and growth. With the sequence of the poplar genome (Tuskan et al., 2006) and multiple studies of the poplar microbiome (Hacquard and Schadt, 2015), poplar can become a model system for studying tree-microbiome interactions. The *Laccaria-Populus* interaction is a well-studied mycorrhizal mutualism at the molecular level (Podila et al., 2009; Aminov, 2011; Larsen et al., 2011; Plett et al., 2015). With comparative genomics studies of the bacteria, mycorrhizae, and yeast associated with *Populus*, an understanding of common themes in plant-mutualist interactions may emerge.

## Data access

The genome version discussed in this paper is the *Rhodotorula graminis* strain WP1 v1.1. Assembled scaffolds and all predicted genes and annotations are available at JGI fungal genome portal Mycocosm (http://genome.jgi.doe.gov/Rhoba1_1) and deposited to GenBank under accession JTAO00000000.

### Conflict of interest statement

The authors declare that the research was conducted in the absence of any commercial or financial relationships that could be construed as a potential conflict of interest.
